# Supplementary material for: Initiations of safer supply hydromorphone increased during the COVID-19 pandemic in Ontario: An interrupted time series analysis
Source: PLoS One. 2023 Dec 19;18(12):e0295145. doi: 10.1371/journal.pone.0295145 (PMC10729949; doi:10.1371/journal.pone.0295145)
Supplement: S1 Appendix — (DOCX) [file pone.0295145.s001.docx]

We estimated the following equation:

$$\ln\left( Y_{t} \right)=\beta_{0}+\beta_{1}t+\beta_{2}X_{t}+\beta_{3}X_{t}(t-t^{*})+\epsilon_{t}$$

Where:

$Y_{t}$ is the number of events in the *t^th^* time period, *t* = 0, … , *N*, where *N* is the number of time periods in the analysis. For Poisson and negative binomial models, *Y* is a count variable and the natural logarithm of the count is modeled

$\beta_{0}$ is a constant term indicating the natural log of the number of initiations at the start of the analysis (*t* = 0)

$\beta_{1}$ is the coefficient representing the increase in the natural log of the number of initiations in each time period

$t$is the number of time periods that have elapsed

$\beta_{2}$ is the coefficient for the level change at the time of the intervention (COVID-19 declaration of emergency)

$X_{t}$ is an indicator variable for the *t^th^* time period, equal to 0 for time periods prior and 1 for periods after the intervention date

$\beta_{3}$ is the coefficient for the change in trend for time periods after the intervention

$t^{*}$ is the time period at the start of the intervention

$X_{t}T_{t}$ is the interaction term for the *t^th^* time period between the indicator variable and the number of elapsed time periods since the intervention.

$\epsilon_{t}$ is the error term and can be specified using a robust variance estimator to account for overdispersion of the data and, if needed, as a heteroskedasticity- and autocorrelation-consistent variance estimator to account for autocorrelated data.

In our base model, we considered the intervention to be the start of the pandemic and the intervention date to be March 17, 2020, the date that the pandemic declaration was declared.

In this model, the exponentiated coefficients are easier to interpret; for example, $e^{\beta_{0}}$ represents the baseline incidence rate and $e^{\beta_{1}}$ represents the incidence rate ratio per 28 day period prior to the pandemic. The exponentiated $\beta_{2}$ and $\beta_{3}$ coefficients represent the incidence rate ratios at and after the intervention. Since time is cumulative, the incidence rate at any time during the analysis should consider which coefficients are operative. For example, the incidence rate during the pandemic is equal to $e^{\beta_{0}+\beta_{1}t+\beta_{2}{+\beta}_{3}(t-t^{*})}$, which is algebraically equivalent to $e^{\beta_{0}}\times e^{\beta_{1}t}\times e^{\beta_{2}}\times e^{\beta_{3}(t-t^{*})}$.

To account for seasonality in the data, we added 2 pairs of Fourier terms:

$$\ln\left( Y_{t}|X_{t}, T_{t} \right)=\beta_{0}+\beta_{1}T_{t}+\beta_{2}X_{t}+\beta_{3}X_{t}T_{t}+\sum_{j=1}^{2} s_{j}sin(2j\pi u){+\sum_{j=1}^{2} c_{j}\cos(2j\pi u)+ \epsilon}_{t}$$

Where $u$ represents a frequency.

Fourier terms allow for flexible modeling of functions that fluctuate regularly over time.
