# Supplementary figures and images for: Initiations of safer supply hydromorphone increased during the COVID-19 pandemic in Ontario: An interrupted time series analysis
Source: PLoS One. 2023 Dec 19;18(12):e0295145. doi: 10.1371/journal.pone.0295145 (PMC10729949; doi:10.1371/journal.pone.0295145)

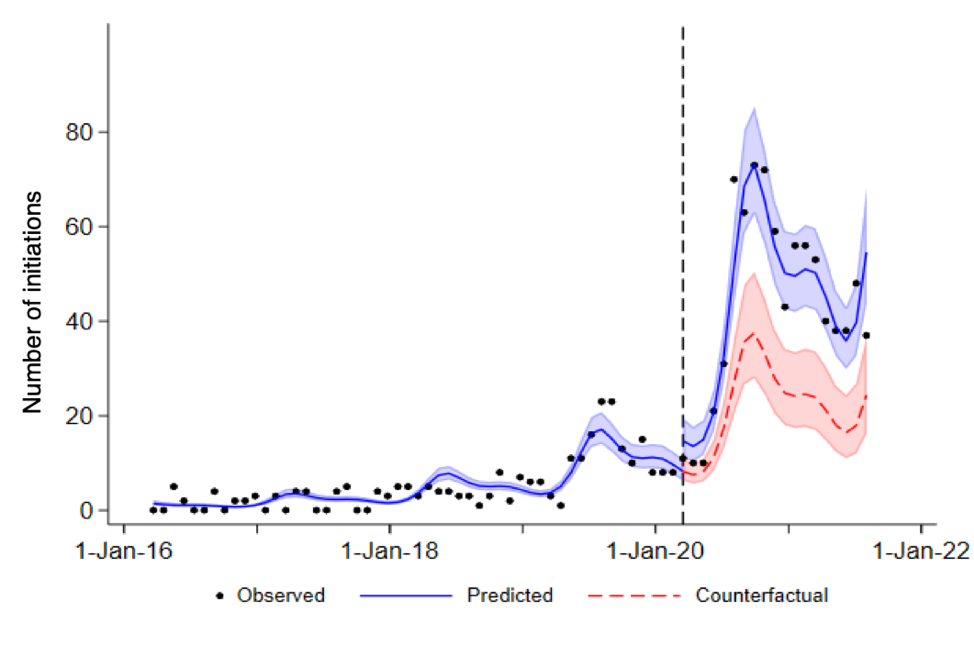

Supplement: S1 Fig — Dots represent the number of safer supply hydromorphone dispensations per 28-day period between early 2016 and late 2021 in Ontario. The vertical dashed line indicates March 17, 2020, the date that Ontario declared an emergency due to the COIVD-19 pandemic. The solid blue line indicates predictions from the best-fitting negative binomial regression model and light blue shading indicates confidence bounds around these estimates. The dashed red line indicates the estimated number of dispensations if the pre-pandemic trends had continued during the pandemic (i.e., the counterfactual scenario) and light red shading indicates confidence bounds around these estimates. (TIFF) [file pone.0295145.s003.tiff]

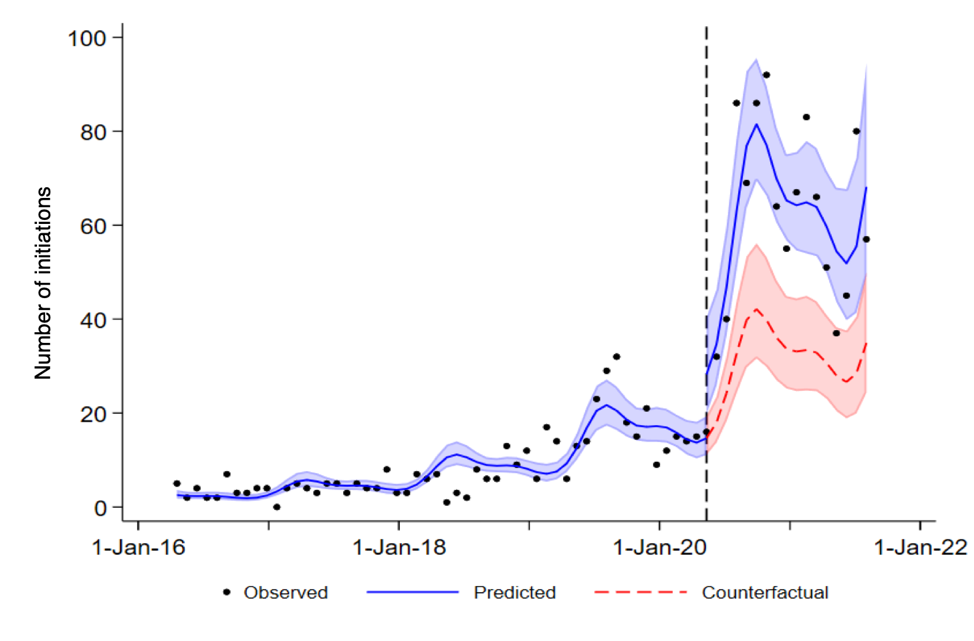

Supplement: S2 Fig — Dots represent the number of safer supply hydromorphone dispensations per 28-day period between early 2016 and late 2021 in Ontario. The vertical dashed line indicates May 12, 2020, the alternate change date. The solid blue line indicates predictions from the best-fitting negative binomial regression model and light blue shading indicates confidence bounds around these estimates. The dashed red line indicates the estimated number of dispensations if the pre-pandemic trends had continued during the pandemic (i.e., the counterfactual scenario) and light red shading indicates confidence bounds around these estimates. (TIFF) [file pone.0295145.s004.tiff]
