# Supplementary material for: Initiations of safer supply hydromorphone increased during the COVID-19 pandemic in Ontario: An interrupted time series analysis
Source: PLoS One. 2023 Dec 19;18(12):e0295145. doi: 10.1371/journal.pone.0295145 (PMC10729949; doi:10.1371/journal.pone.0295145)
Supplement: S1 Table — (DOCX) [file pone.0295145.s005.docx]

**S1 Table. Diagnostic codes used to define aspects of cohort definition and descriptive variables**

| **Characteristic** | **Database** | **Codes** |
| --- | --- | --- |
| **Opioid Use Disorder** | | |
|  | CIHI-DAD, NACRS, OMHRS | **ICD-10:** F11 |
|  | OHIP | **Fee codes:** K682, K683, K684  **Diagnosis code:** 304 **DSM Codes :** 30400 and 30550 |
| **Opioid-related Overdose** | | |
|  | CIHI-DAD, NACRS | **ICD-10:** T40.0-T40.4, T40.6 |
| **Palliative Care** | | |
|  | OHIP | **Fee codes:** A945, B998, C945, C882, C982, K023, W872, W882, W972, W982 |
| **Cancer Diagnosis or**  **Treatment** | | |
|  | OHIP | **Fee codes:** G281, G339, G345, G359, G381, G382, X310, X311, X312, X313  **Diagnosis code:** Exclude 714 or 555 |
| **Alcohol Use Disorder** | | |
|  | CIHI-DAD, NACRS | **ICD-10:** F10, K70, G31.2, G62.1 G72.1 I42.6 K29.2 K70.1 K70.4 K70.9 K86.0 Z50.2 Z71.4 Z86.40 |
|  | OHIP | **Diagnosis code:** 291, 303 |
| **Infective Endocarditis** |  |  |
|  | CIHI-DAD, NACRS | **ICD-10:** I33.0 (Acute and subacute infective endocarditis), I33.9 (Acute and subacute endocarditis, unspecified),I38 (Endocarditis, valve unspecified), I39 (Endocarditis and heart valve disorders in diseases classified elsewhere), B37.6 (Candidal endocarditis) |
| **Osteomyelitis or Discitis** | | |
|  | CIHI-DAD, NACRS | **ICD-10:** M86 (Osteomyelitis), M00 (Osteomyelitis or septic arthritis), G06.1 (Epidural abscess), M46.2 (Osteomyelitis of vertebra), M46.3 (Infection of intervertebral disc [pyogenic]), M46.4 (Discitis, unspecified), M46.5 (Osteomyelitis or septic arthritis) |
| **Skin and Soft Tissue Infections** | | |
|  | CIHI-DAD, NACRS | **ICD-10:** L03 (Cellulitis), L02 (Cutaneous abscess), M76.2 (Necrotizing fasciitis) |
| CIHI-DAD=Canadian Institutes for Health Information Discharge Abstract Database; NACRS=National Ambulatory Care Reporting System; OMHRS=Ontario Mental Health Reporting System. | | |
