# Supplementary material for: Initiations of safer supply hydromorphone increased during the COVID-19 pandemic in Ontario: An interrupted time series analysis
Source: PLoS One. 2023 Dec 19;18(12):e0295145. doi: 10.1371/journal.pone.0295145 (PMC10729949; doi:10.1371/journal.pone.0295145)
Supplement: S2 Table — (DOCX) [file pone.0295145.s006.docx]

**S2 Table. Full table of results (including constant and Fourier transformation terms) for primary, sex-specific, and sensitivity analyses comparing initiations of safer supply hydromorphone before and during the COVID-19 pandemic**

|  | **Incidence Rate Ratio** | **(95% Confidence Interval)** | **P-value** |
| --- | --- | --- | --- |
| **Baseline model – All individuals** | | |  |
| Pre-pandemic trend | 1.002 | (1.001 to 1.002) | <0.001 |
| Pandemic change | 1.674 | (1.206 to 2.322) | 0.002 |
| Pandemic trend | 1.001 | (1.000 to 1.001) | 0.25 |
| Constant | 2.095 | (1.485 to 2.956) | <0.001 |
| Sine 1 | 0.937 | (0.830 to 1.057) | 0.290 |
| Sine 2 | 1.159 | (1.051 to 1.279) | 0.003 |
| Cosine 1 | 0.655 | (0.582 to 0.737) | <0.001 |
| Cosine 2 | 0.933 | (0.823 to 1.059) | 0.285 |
| **Sex-specific model – Males** | | | |
| Pre-pandemic trend | 1.002 | (1.001 to 1.002) | <0.001 |
| Pandemic change | 1.691 | (1.075 to 2.658) | 0.023 |
| Pandemic trend | 1.000 | (0.999 to 1.002) | 0.66 |
| Constant | 0.957 | (0.531 to 1.724) | 0.883 |
| Sine 1 | 0.974 | (0.816 to 1.161) | 0.767 |
| Sine 2 | 1.211 | (1.057 to 1.387) | 0.006 |
| Cosine 1 | 0.634 | (0.550 to 0.730) | <0.001 |
| Cosine 2 | 0.913 | (0.778 to 1.071) | 0.263 |
| **Sex-specific model – Females** | | | |
| Pre-pandemic trend | 1.002 | (1.001 to 1.003) | <0.001 |
| Pandemic change | 1.289 | (0.912 to 1.821) | 0.15 |
| Pandemic trend | 1.000 | (0.999 to 1.001) | 0.90 |
| Constant | 0.517 | (0.266 to 1.004) | 0.051 |
| Sine 1 | 0.857 | (0.757 to 0.971) | 0.016 |
| Cosine 1 | 0.686 | (0.579 to 0.814) | <0.001 |
| **Sensitivity analysis excluding individuals who received 4mg or 8mg hydromorphone in 14 days prior to index date** | | | |
| Pre-pandemic trend | 1.002 | (1.002 to 1.002) | <0.001 |
| Pandemic change | 1.783 | (1.335 to 2.383) | <0.001 |
| Pandemic trend | 1.001 | (1.000 to 1.001) | 0.25 |
| Constant | 0.891 | (0.608 to 1.306) | 0.56 |
| Sine 1 | 0.919 | (0.828 to 1.019) | 0.11 |
| Sine 2 | 1.252 | (1.134 to 1.383) | <0.001 |
| Cosine 1 | 0.586 | (0.528 to 0.652) | <0.001 |
| Cosine 2 | 0.943 | (0.853 to 1.042) | 0.25 |
| **Sensitivity analysis with alternate intervention date May 12, 2020** | | | |
| Pre-pandemic trend | 1.002 | (1.001 to 1.002) | <0.001 |
| Pandemic change | 1.925 | (1.381 to 2.685) | <0.001 |
| Pandemic trend | 1.000 | (0.999 to 1.001) | 0.955 |
| Constant | 2.109 | (1.521 to 2.923) | <0.001 |
| Sine 1 | 0.955 | (0.848 to 1.076) | 0.45 |
| Sine 2 | 1.149 | (1.037 to 1.272) | 0.008 |
| Cosine 1 | 0.698 | (0.611 to 0.797) | <0.001 |
| Cosine 2 | 0.969 | (0.856 to 1.097) | 0.62 |

All models use an intervention date of May 17, 2020 unless otherwise specified.
